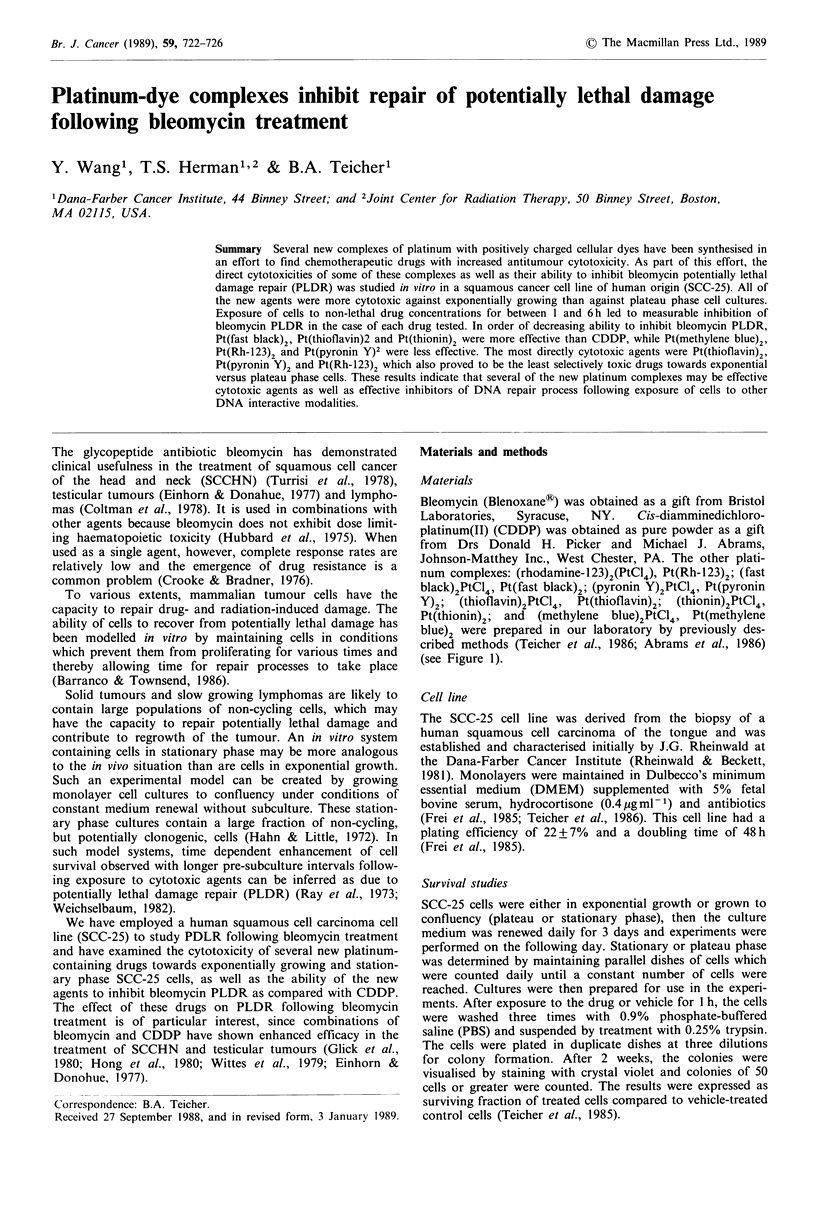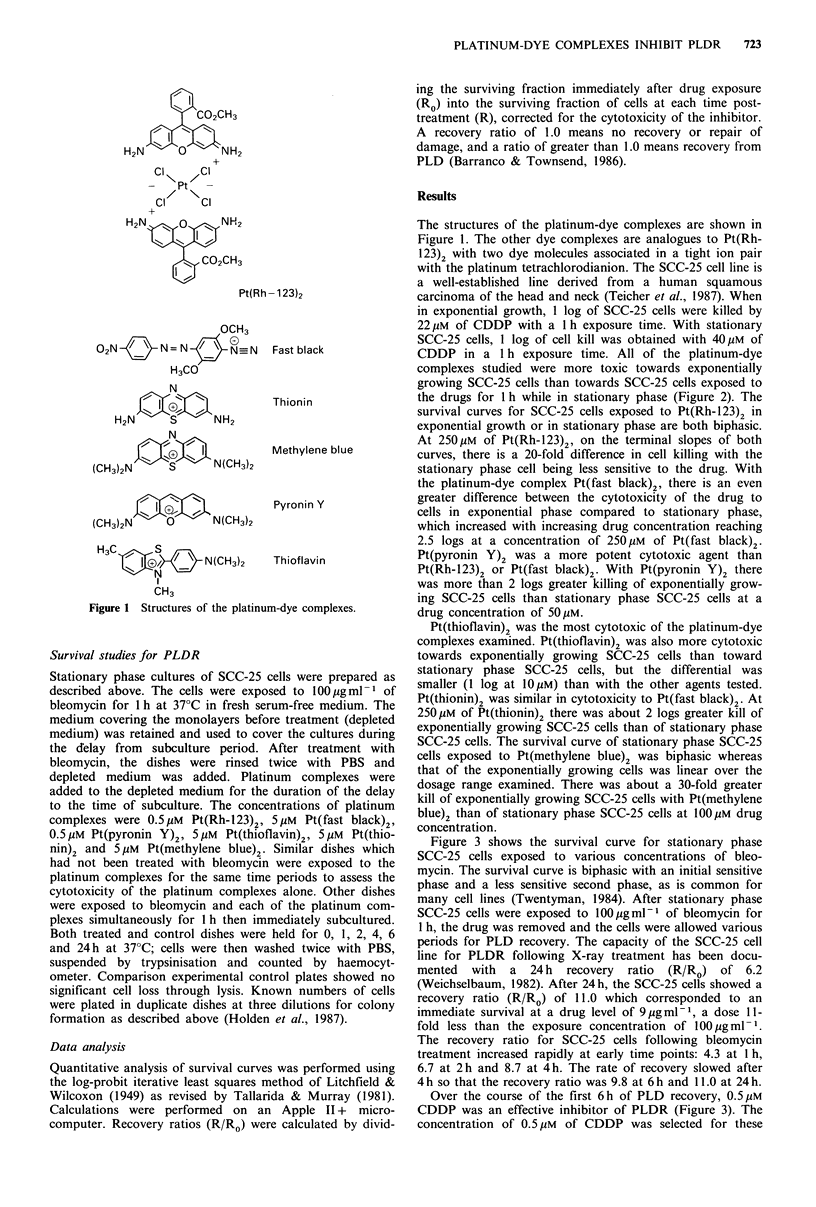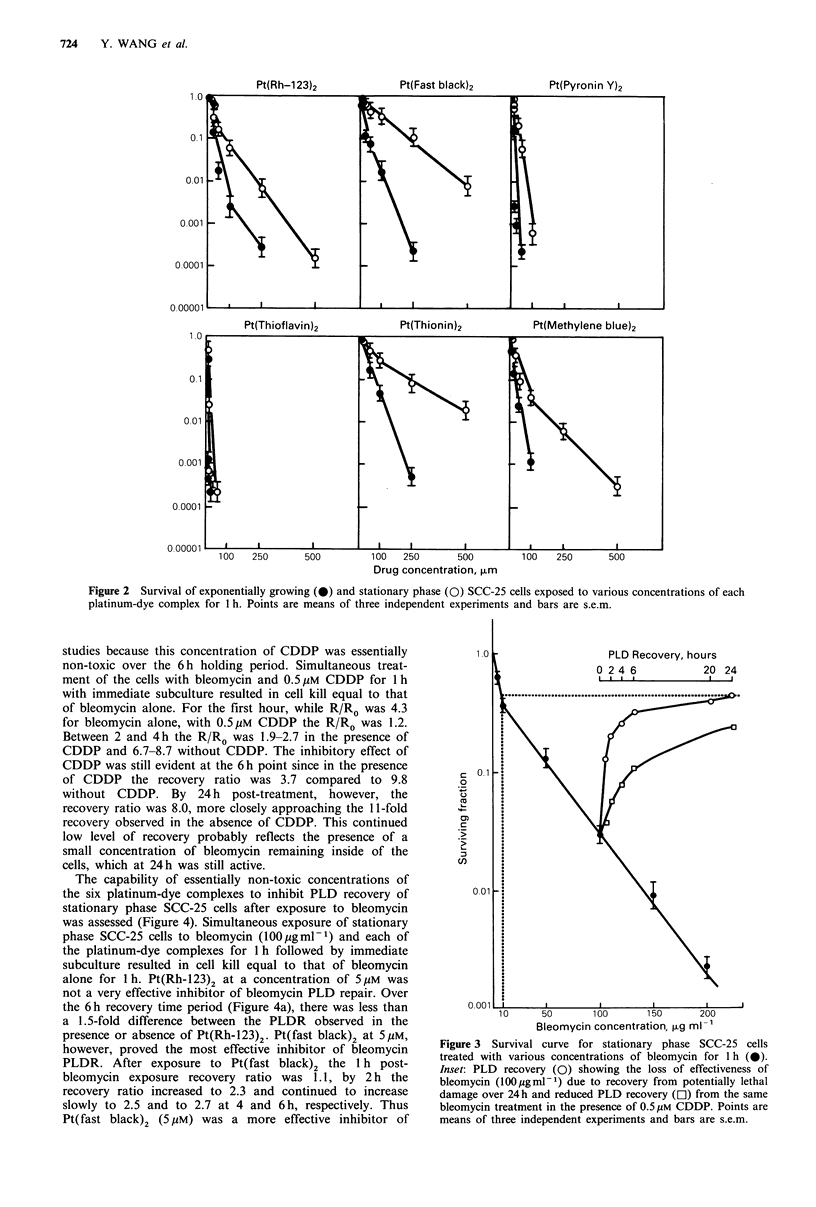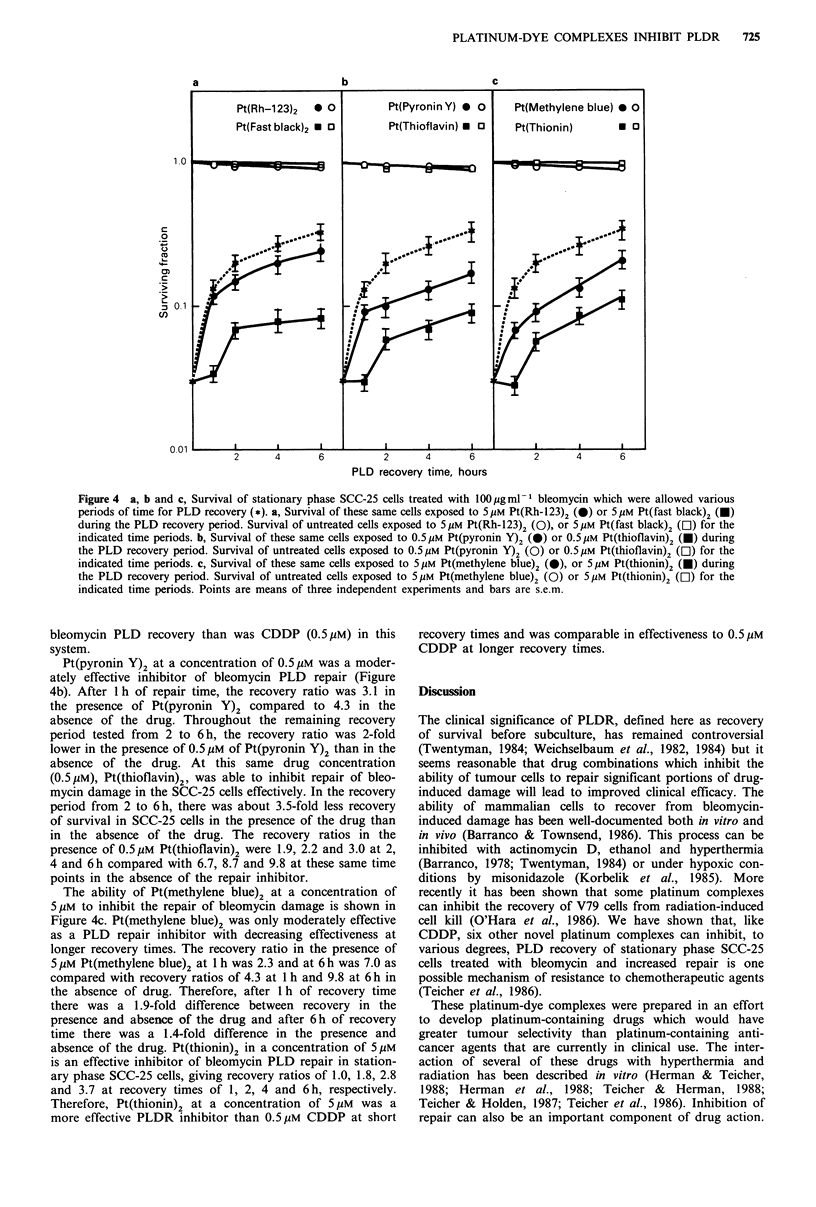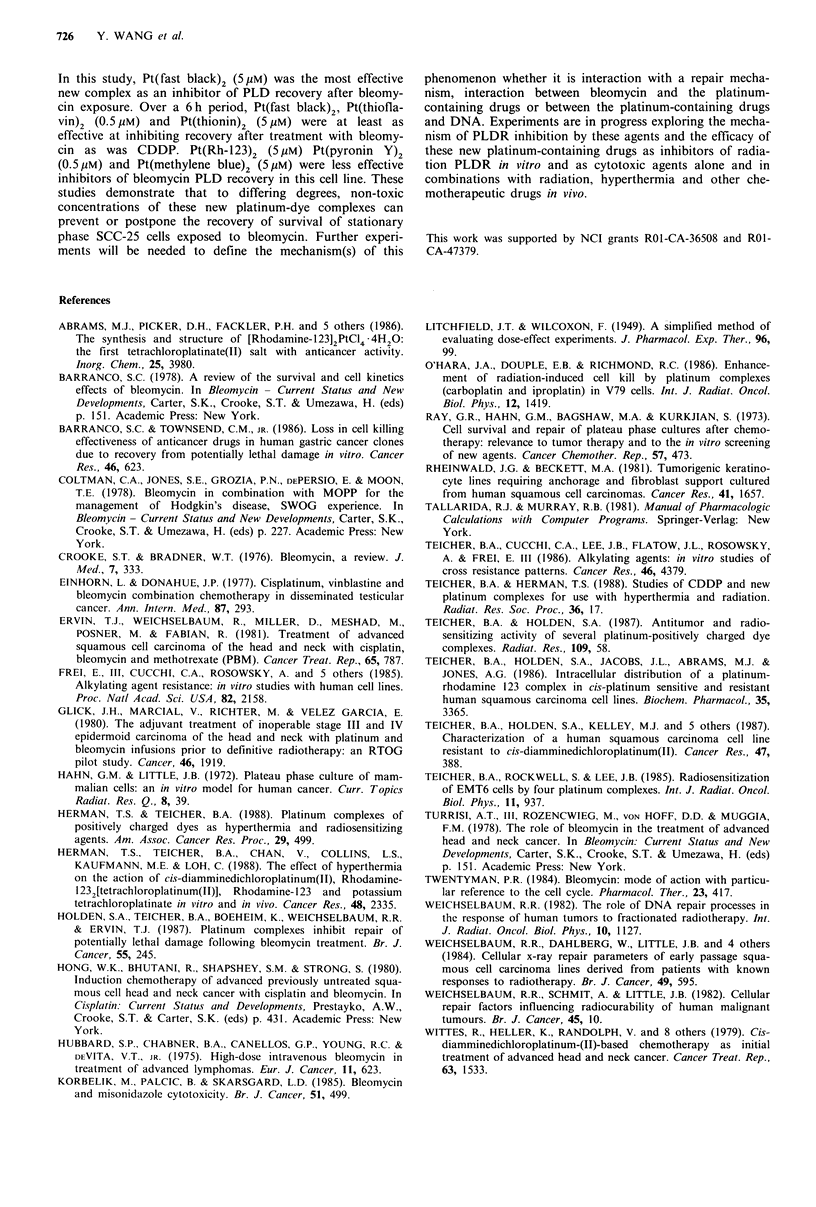# Platinum-dye complexes inhibit repair of potentially lethal damage following bleomycin treatment.

**DOI:** 10.1038/bjc.1989.152

**Published:** 1989-05

**Authors:** Y. Wang, T. S. Herman, B. A. Teicher

**Affiliations:** Dana-Farber Cancer Institute, Boston, MA.

## Abstract

Several new complexes of platinum with positively charged cellular dyes have been synthesised in an effort to find chemotherapeutic drugs with increased antitumour cytotoxicity. As part of this effort, the direct cytotoxicities of some of these complexes as well as their ability to inhibit bleomycin potentially lethal damage repair (PLDR) was studied in vitro in a squamous cancer cell line of human origin (SCC-25). All of the new agents were more cytotoxic against exponentially growing than against plateau phase cell cultures. Exposure of cells to non-lethal drug concentrations for between 1 and 6 h led to measurable inhibition of bleomycin PLDR in the case of each drug tested. In order of decreasing ability to inhibit bleomycin PLDR, Pt(fast black)2, Pt(thioflavin)2 and Pt(thionin)2 were more effective than CDDP, while Pt(methylene blue)2, Pt(Rh-123)2 and Pt(pyronin Y)2 were less effective. The most directly cytotoxic agents were Pt(thioflavin)2, Pt(pyronin Y)2 and Pt(Rh-123)2 which also proved to be the least selectively toxic drugs towards exponential versus plateau phase cells. These results indicate that several of the new platinum complexes may be effective cytotoxic agents as well as effective inhibitors of DNA repair process following exposure of cells to other DNA interactive modalities.


*Br. J. Cancer* (1989), **59**, 722–726 © The Macmillan Press Ltd., 1989

### Platinum-dye complexes inhibit repair of potentially lethal damage following bleomycin treatment

Y. Wang[1], T.S. Herman[1,2] & B.A. Teicher[1]

[1]*Dana-Farber Cancer Institute, 44 Binney Street; and* [2]*Joint Center for Radiation Therapy, 50 Binney Street, Boston, MA 02115, USA.*

**Summary** Several new complexes of platinum with positively charged cellular dyes have been synthesised in an effort to find chemotherapeutic drugs with increased antitumour cytotoxicity. As part of this effort, the direct cytotoxicities of some of these complexes as well as their ability to inhibit bleomycin potentially lethal damage repair (PLDR) was studied *in vitro* in a squamous cancer cell line of human origin (SCC-25). All of the new agents were more cytotoxic against exponentially growing than against plateau phase cell cultures. Exposure of cells to non-lethal drug concentrations for between 1 and 6 h led to measurable inhibition of bleomycin PLDR in the case of each drug tested. In order of decreasing ability to inhibit bleomycin PLDR, Pt(fast black)$_2$, Pt(thioflavin)2 and Pt(thionin)$_2$ were more effective than CDDP, while Pt(methylene blue)$_2$, Pt(Rh-123)$_2$ and Pt(pyronin Y)$^2$ were less effective. The most directly cytotoxic agents were Pt(thioflavin)$_2$, Pt(pyronin Y)$_2$ and Pt(Rh-123)$_2$ which also proved to be the least selectively toxic drugs towards exponential versus plateau phase cells. These results indicate that several of the new platinum complexes may be effective cytotoxic agents as well as effective inhibitors of DNA repair process following exposure of cells to other DNA interactive modalities.

The glycopeptide antibiotic bleomycin has demonstrated clinical usefulness in the treatment of squamous cell cancer of the head and neck (SCCHN) (Turrisi *et al.*, 1978), testicular tumours (Einhorn & Donahue, 1977) and lymphomas (Coltman *et al.*, 1978). It is used in combinations with other agents because bleomycin does not exhibit dose limiting haematopoietic toxicity (Hubbard *et al.*, 1975). When used as a single agent, however, complete response rates are relatively low and the emergence of drug resistance is a common problem (Crooke & Bradner, 1976).

To various extents, mammalian tumour cells have the capacity to repair drug- and radiation-induced damage. The ability of cells to recover from potentially lethal damage has been modelled *in vitro* by maintaining cells in conditions which prevent them from proliferating for various times and thereby allowing time for repair processes to take place (Barranco & Townsend, 1986).

Solid tumours and slow growing lymphomas are likely to contain large populations of non-cycling cells, which may have the capacity to repair potentially lethal damage and contribute to regrowth of the tumour. An *in vitro* system containing cells in stationary phase may be more analogous to the *in vivo* situation than are cells in exponential growth. Such an experimental model can be created by growing monolayer cell cultures to confluency under conditions of constant medium renewal without subculture. These stationary phase cultures contain a large fraction of non-cycling, but potentially clonogenic, cells (Hahn & Little, 1972). In such model systems, time dependent enhancement of cell survival observed with longer pre-subculture intervals following exposure to cytotoxic agents can be inferred as due to potentially lethal damage repair (PLDR) (Ray *et al.*, 1973; Weichselbaum, 1982).

We have employed a human squamous cell carcinoma cell line (SCC-25) to study PDLR following bleomycin treatment and have examined the cytotoxicity of several new platinum-containing drugs towards exponentially growing and stationary phase SCC-25 cells, as well as the ability of the new agents to inhibit bleomycin PLDR as compared with CDDP. The effect of these drugs on PLDR following bleomycin treatment is of particular interest, since combinations of bleomycin and CDDP have shown enhanced efficacy in the treatment of SCCHN and testicular tumours (Glick *et al.*, 1980; Hong *et al.*, 1980; Wittes *et al.*, 1979; Einhorn & Donohue, 1977).

Correspondence: B.A. Teicher.
Received 27 September 1988, and in revised form, 3 January 1989.

### Materials and methods

### *Materials*

Bleomycin (Blenoxane®) was obtained as a gift from Bristol Laboratories, Syracuse, NY. *Cis*-diamminedichloroplatinum(II) (CDDP) was obtained as pure powder as a gift from Drs Donald H. Picker and Michael J. Abrams, Johnson-Matthey Inc., West Chester, PA. The other platinum complexes: (rhodamine-123)$_2$($PtCl_4$), Pt(Rh-123)$_2$; (fast black)$_2$$PtCl_4$, Pt(fast black)$_2$; (pyronin Y)$_2$$PtCl_4$, Pt(pyronin Y)$_2$; (thioflavin)$_2$$PtCl_4$, Pt(thioflavin)$_2$; (thionin)$_2$$PtCl_4$, Pt(thionin)$_2$; and (methylene blue)$_2$$PtCl_4$, Pt(methylene blue)$_2$ were prepared in our laboratory by previously described methods (Teicher *et al.*, 1986; Abrams *et al.*, 1986) (see Figure 1).

### *Cell line*

The SCC-25 cell line was derived from the biopsy of a human squamous cell carcinoma of the tongue and was established and characterised initially by J.G. Rheinwald at the Dana-Farber Cancer Institute (Rheinwald & Beckett, 1981). Monolayers were maintained in Dulbecco's minimum essential medium (DMEM) supplemented with 5% fetal bovine serum, hydrocortisone (0.4 $\mu g\,ml^{-1}$) and antibiotics (Frei *et al.*, 1985; Teicher *et al.*, 1986). This cell line had a plating efficiency of $22 \pm 7\%$ and a doubling time of 48 h (Frei *et al.*, 1985).

### *Survival studies*

SCC-25 cells were either in exponential growth or grown to confluency (plateau or stationary phase), then the culture medium was renewed daily for 3 days and experiments were performed on the following day. Stationary or plateau phase was determined by maintaining parallel dishes of cells which were counted daily until a constant number of cells were reached. Cultures were then prepared for use in the experiments. After exposure to the drug or vehicle for 1 h, the cells were washed three times with 0.9% phosphate-buffered saline (PBS) and suspended by treatment with 0.25% trypsin. The cells were plated in duplicate dishes at three dilutions for colony formation. After 2 weeks, the colonies were visualised by staining with crystal violet and colonies of 50 cells or greater were counted. The results were expressed as surviving fraction of treated cells compared to vehicle-treated control cells (Teicher *et al.*, 1985).

**Figure 1** Structures of the platinum-dye complexes.

*Survival studies for PLDR*

Stationary phase cultures of SCC-25 cells were prepared as described above. The cells were exposed to $100 \mu g ml^{-1}$ of bleomycin for 1 h at 37°C in fresh serum-free medium. The medium covering the monolayers before treatment (depleted medium) was retained and used to cover the cultures during the delay from subculture period. After treatment with bleomycin, the dishes were rinsed twice with PBS and depleted medium was added. Platinum complexes were added to the depleted medium for the duration of the delay to the time of subculture. The concentrations of platinum complexes were 0.5 μM Pt(Rh-123)$_2$, 5 μM Pt(fast black)$_2$, 0.5 μM Pt(pyronin Y)$_2$, 5 μM Pt(thioflavin)$_2$, 5 μM Pt(thionin)$_2$ and 5 μM Pt(methylene blue)$_2$. Similar dishes which had not been treated with bleomycin were exposed to the platinum complexes for the same time periods to assess the cytotoxicity of the platinum complexes alone. Other dishes were exposed to bleomycin and each of the platinum complexes simultaneously for 1 h then immediately subcultured. Both treated and control dishes were held for 0, 1, 2, 4, 6 and 24 h at 37°C; cells were then washed twice with PBS, suspended by trypsinisation and counted by haemocytometer. Comparison experimental control plates showed no significant cell loss through lysis. Known numbers of cells were plated in duplicate dishes at three dilutions for colony formation as described above (Holden *et al.*, 1987).

*Data analysis*

Quantitative analysis of survival curves was performed using the log-probit iterative least squares method of Litchfield & Wilcoxon (1949) as revised by Tallarida & Murray (1981). Calculations were performed on an Apple II+ microcomputer. Recovery ratios ($R/R_0$) were calculated by dividing the surviving fraction immediately after drug exposure ($R_0$) into the surviving fraction of cells at each time post-treatment (R), corrected for the cytotoxicity of the inhibitor. A recovery ratio of 1.0 means no recovery or repair of damage, and a ratio of greater than 1.0 means recovery from PLD (Barranco & Townsend, 1986).

### Results

The structures of the platinum-dye complexes are shown in Figure 1. The other dye complexes are analogues to Pt(Rh-123)$_2$ with two dye molecules associated in a tight ion pair with the platinum tetrachlorodianion. The SCC-25 cell line is a well-established line derived from a human squamous carcinoma of the head and neck (Teicher *et al.*, 1987). When in exponential growth, 1 log of SCC-25 cells were killed by 22 μM of CDDP with a 1 h exposure time. With stationary SCC-25 cells, 1 log of cell kill was obtained with 40 μM of CDDP in a 1 h exposure time. All of the platinum-dye complexes studied were more toxic towards exponentially growing SCC-25 cells than towards SCC-25 cells exposed to the drugs for 1 h while in stationary phase (Figure 2). The survival curves for SCC-25 cells exposed to Pt(Rh-123)$_2$ in exponential growth or in stationary phase are both biphasic. At 250 μM of Pt(Rh-123)$_2$, on the terminal slopes of both curves, there is a 20-fold difference in cell killing with the stationary phase cell being less sensitive to the drug. With the platinum-dye complex Pt(fast black)$_2$, there is an even greater difference between the cytotoxicity of the drug to cells in exponential phase compared to stationary phase, which increased with increasing drug concentration reaching 2.5 logs at a concentration of 250 μM of Pt(fast black)$_2$. Pt(pyronin Y)$_2$ was a more potent cytotoxic agent than Pt(Rh-123)$_2$ or Pt(fast black)$_2$. With Pt(pyronin Y)$_2$ there was more than 2 logs greater killing of exponentially growing SCC-25 cells than stationary phase SCC-25 cells at a drug concentration of 50 μM.

Pt(thioflavin)$_2$ was the most cytotoxic of the platinum-dye complexes examined. Pt(thioflavin)$_2$ was also more cytotoxic towards exponentially growing SCC-25 cells than toward stationary phase SCC-25 cells, but the differential was smaller (1 log at 10 μM) than with the other agents tested. Pt(thionin)$_2$ was similar in cytotoxicity to Pt(fast black)$_2$. At 250 μM of Pt(thionin)$_2$ there was about 2 logs greater kill of exponentially growing SCC-25 cells than of stationary phase SCC-25 cells. The survival curve of stationary phase SCC-25 cells exposed to Pt(methylene blue)$_2$ was biphasic whereas that of the exponentially growing cells was linear over the dosage range examined. There was about a 30-fold greater kill of exponentially growing SCC-25 cells with Pt(methylene blue)$_2$ than of stationary phase SCC-25 cells at 100 μM drug concentration.

Figure 3 shows the survival curve for stationary phase SCC-25 cells exposed to various concentrations of bleomycin. The survival curve is biphasic with an initial sensitive phase and a less sensitive second phase, as is common for many cell lines (Twentyman, 1984). After stationary phase SCC-25 cells were exposed to $100 \mu g ml^{-1}$ of bleomycin for 1 h, the drug was removed and the cells were allowed various periods for PLD recovery. The capacity of the SCC-25 cell line for PLDR following X-ray treatment has been documented with a 24 h recovery ratio ($R/R_0$) of 6.2 (Weichselbaum, 1982). After 24 h, the SCC-25 cells showed a recovery ratio ($R/R_0$) of 11.0 which corresponded to an immediate survival at a drug level of $9 \mu g ml^{-1}$, a dose 11-fold less than the exposure concentration of $100 \mu g ml^{-1}$. The recovery ratio for SCC-25 cells following bleomycin treatment increased rapidly at early time points: 4.3 at 1 h, 6.7 at 2 h and 8.7 at 4 h. The rate of recovery slowed after 4 h so that the recovery ratio was 9.8 at 6 h and 11.0 at 24 h.

Over the course of the first 6 h of PLD recovery, 0.5 μM CDDP was an effective inhibitor of PLDR (Figure 3). The concentration of 0.5 μM of CDDP was selected for these

**Figure 2** Survival of exponentially growing (●) and stationary phase (○) SCC-25 cells exposed to various concentrations of each platinum-dye complex for 1 h. Points are means of three independent experiments and bars are s.e.m.

studies because this concentration of CDDP was essentially non-toxic over the 6 h holding period. Simultaneous treatment of the cells with bleomycin and 0.5 μM CDDP for 1 h with immediate subculture resulted in cell kill equal to that of bleomycin alone. For the first hour, while $R/R_0$ was 4.3 for bleomycin alone, with 0.5 μM CDDP the $R/R_0$ was 1.2. Between 2 and 4 h the $R/R_0$ was 1.9–2.7 in the presence of CDDP and 6.7–8.7 without CDDP. The inhibitory effect of CDDP was still evident at the 6 h point since in the presence of CDDP the recovery ratio was 3.7 compared to 9.8 without CDDP. By 24 h post-treatment, however, the recovery ratio was 8.0, more closely approaching the 11-fold recovery observed in the absence of CDDP. This continued low level of recovery probably reflects the presence of a small concentration of bleomycin remaining inside of the cells, which at 24 h was still active.

The capability of essentially non-toxic concentrations of the six platinum-dye complexes to inhibit PLD recovery of stationary phase SCC-25 cells after exposure to bleomycin was assessed (Figure 4). Simultaneous exposure of stationary phase SCC-25 cells to bleomycin (100 $\mu g\,ml^{-1}$) and each of the platinum-dye complexes for 1 h followed by immediate subculture resulted in cell kill equal to that of bleomycin alone for 1 h. $Pt(Rh\text{-}123)_2$ at a concentration of 5 μM was not a very effective inhibitor of bleomycin PLD repair. Over the 6 h recovery time period (Figure 4a), there was less than a 1.5-fold difference between the PLDR observed in the presence or absence of $Pt(Rh\text{-}123)_2$. $Pt(fast\ black)_2$ at 5 μM, however, proved the most effective inhibitor of bleomycin PLDR. After exposure to $Pt(fast\ black)_2$ the 1 h post-bleomycin exposure recovery ratio was 1.1, by 2 h the recovery ratio increased to 2.3 and continued to increase slowly to 2.5 and to 2.7 at 4 and 6 h, respectively. Thus $Pt(fast\ black)_2$ (5 μM) was a more effective inhibitor of

**Figure 3** Survival curve for stationary phase SCC-25 cells treated with various concentrations of bleomycin for 1 h (●). *Inset*: PLD recovery (○) showing the loss of effectiveness of bleomycin (100 $\mu g\,ml^{-1}$) due to recovery from potentially lethal damage over 24 h and reduced PLD recovery (□) from the same bleomycin treatment in the presence of 0.5 μM CDDP. Points are means of three independent experiments and bars are s.e.m.

**Figure 4** **a**, **b** and **c**, Survival of stationary phase SCC-25 cells treated with 100 $\mu g\,ml^{-1}$ bleomycin which were allowed various periods of time for PLD recovery (*). **a**, Survival of these same cells exposed to 5 $\mu$M Pt(Rh-123)$_2$ (●) or 5 $\mu$M Pt(fast black)$_2$ (■) during the PLD recovery period. Survival of untreated cells exposed to 5 $\mu$M Pt(Rh-123)$_2$ (○), or 5 $\mu$M Pt(fast black)$_2$ (□) for the indicated time periods. **b**, Survival of these same cells exposed to 0.5 $\mu$M Pt(pyronin Y)$_2$ (●) or 0.5 $\mu$M Pt(thioflavin)$_2$ (■) during the PLD recovery period. Survival of untreated cells exposed to 0.5 $\mu$M Pt(pyronin Y)$_2$ (○) or 0.5 $\mu$M Pt(thioflavin)$_2$ (□) for the indicated time periods. **c**, Survival of these same cells exposed to 5 $\mu$M Pt(methylene blue)$_2$ (●), or 5 $\mu$M Pt(thionin)$_2$ (■) during the PLD recovery period. Survival of untreated cells exposed to 5 $\mu$M Pt(methylene blue)$_2$ (○) or 5 $\mu$M Pt(thionin)$_2$ (□) for the indicated time periods. Points are means of three independent experiments and bars are s.e.m.

bleomycin PLD recovery than was CDDP (0.5 $\mu$M) in this system.

Pt(pyronin Y)$_2$ at a concentration of 0.5 $\mu$M was a moderately effective inhibitor of bleomycin PLD repair (Figure 4b). After 1 h of repair time, the recovery ratio was 3.1 in the presence of Pt(pyronin Y)$_2$ compared to 4.3 in the absence of the drug. Throughout the remaining recovery period tested from 2 to 6 h, the recovery ratio was 2-fold lower in the presence of 0.5 $\mu$M of Pt(pyronin Y)$_2$ than in the absence of the drug. At this same drug concentration (0.5 $\mu$M), Pt(thioflavin)$_2$, was able to inhibit repair of bleomycin damage in the SCC-25 cells effectively. In the recovery period from 2 to 6 h, there was about 3.5-fold less recovery of survival in SCC-25 cells in the presence of the drug than in the absence of the drug. The recovery ratios in the presence of 0.5 $\mu$M Pt(thioflavin)$_2$ were 1.9, 2.2 and 3.0 at 2, 4 and 6 h compared with 6.7, 8.7 and 9.8 at these same time points in the absence of the repair inhibitor.

The ability of Pt(methylene blue)$_2$ at a concentration of 5 $\mu$M to inhibit the repair of bleomycin damage is shown in Figure 4c. Pt(methylene blue)$_2$ was only moderately effective as a PLD repair inhibitor with decreasing effectiveness at longer recovery times. The recovery ratio in the presence of 5 $\mu$M Pt(methylene blue)$_2$ at 1 h was 2.3 and at 6 h was 7.0 as compared with recovery ratios of 4.3 at 1 h and 9.8 at 6 h in the absence of drug. Therefore, after 1 h of recovery time there was a 1.9-fold difference between recovery in the presence and absence of the drug and after 6 h of recovery time there was a 1.4-fold difference in the presence and absence of the drug. Pt(thionin)$_2$ in a concentration of 5 $\mu$M is an effective inhibitor of bleomycin PLD repair in stationary phase SCC-25 cells, giving recovery ratios of 1.0, 1.8, 2.8 and 3.7 at recovery times of 1, 2, 4 and 6 h, respectively. Therefore, Pt(thionin)$_2$ at a concentration of 5 $\mu$M was a more effective PLDR inhibitor than 0.5 $\mu$M CDDP at short recovery times and was comparable in effectiveness to 0.5 $\mu$M CDDP at longer recovery times.

### Discussion

The clinical significance of PLDR, defined here as recovery of survival before subculture, has remained controversial (Twentyman, 1984; Weichselbaum *et al.*, 1982, 1984) but it seems reasonable that drug combinations which inhibit the ability of tumour cells to repair significant portions of drug-induced damage will lead to improved clinical efficacy. The ability of mammalian cells to recover from bleomycin-induced damage has been well-documented both *in vitro* and *in vivo* (Barranco & Townsend, 1986). This process can be inhibited with actinomycin D, ethanol and hyperthermia (Barranco, 1978; Twentyman, 1984) or under hypoxic conditions by misonidazole (Korbelik *et al.*, 1985). More recently it has been shown that some platinum complexes can inhibit the recovery of V79 cells from radiation-induced cell kill (O'Hara *et al.*, 1986). We have shown that, like CDDP, six other novel platinum complexes can inhibit, to various degrees, PLD recovery of stationary phase SCC-25 cells treated with bleomycin and increased repair is one possible mechanism of resistance to chemotherapeutic agents (Teicher *et al.*, 1986).

These platinum-dye complexes were prepared in an effort to develop platinum-containing drugs which would have greater tumour selectivity than platinum-containing anticancer agents that are currently in clinical use. The interaction of several of these drugs with hyperthermia and radiation has been described *in vitro* (Herman & Teicher, 1988; Herman *et al.*, 1988; Teicher & Herman, 1988; Teicher & Holden, 1987; Teicher *et al.*, 1986). Inhibition of repair can also be an important component of drug action.

In this study, Pt(fast black)$_2$ (5 $\mu$M) was the most effective new complex as an inhibitor of PLD recovery after bleomycin exposure. Over a 6 h period, Pt(fast black)$_2$, Pt(thioflavin)$_2$ (0.5 $\mu$M) and Pt(thionin)$_2$ (5 $\mu$M) were at least as effective at inhibiting recovery after treatment with bleomycin as was CDDP. Pt(Rh-123)$_2$ (5 $\mu$M) Pt(pyronin Y)$_2$ (0.5 $\mu$M) and Pt(methylene blue)$_2$ (5 $\mu$M) were less effective inhibitors of bleomycin PLD recovery in this cell line. These studies demonstrate that to differing degrees, non-toxic concentrations of these new platinum-dye complexes can prevent or postpone the recovery of survival of stationary phase SCC-25 cells exposed to bleomycin. Further experiments will be needed to define the mechanism(s) of this phenomenon whether it is interaction with a repair mechanism, interaction between bleomycin and the platinum-containing drugs or between the platinum-containing drugs and DNA. Experiments are in progress exploring the mechanism of PLDR inhibition by these agents and the efficacy of these new platinum-containing drugs as inhibitors of radiation PLDR *in vitro* and as cytotoxic agents alone and in combinations with radiation, hyperthermia and other chemotherapeutic drugs *in vivo*.

This work was supported by NCI grants R01-CA-36508 and R01-CA-47379.

### References

ABRAMS, M.J., PICKER, D.H., FACKLER, P.H. and 5 others (1986). The synthesis and structure of [Rhodamine-123]$_2$PtCl$_4$·4H$_2$O: the first tetrachloroplatinate(II) salt with anticancer activity. *Inorg. Chem.*, **25**, 3980.

BARRANCO, S.C. (1978). A review of the survival and cell kinetics effects of bleomycin. In *Bleomycin – Current Status and New Developments*, Carter, S.K., Crooke, S.T. & Umezawa, H. (eds) p. 151. Academic Press: New York.

BARRANCO, S.C. & TOWNSEND, C.M., JR. (1986). Loss in cell killing effectiveness of anticancer drugs in human gastric cancer clones due to recovery from potentially lethal damage *in vitro*. *Cancer Res.*, **46**, 623.

COLTMAN, C.A., JONES, S.E., GROZIA, P.N., DEPERSIO, E. & MOON, T.E. (1978). Bleomycin in combination with MOPP for the management of Hodgkin's disease, SWOG experience. In *Bleomycin – Current Status and New Developments*, Carter, S.K., Crooke, S.T. & Umezawa, H. (eds) p. 227. Academic Press: New York.

CROOKE, S.T. & BRADNER, W.T. (1976). Bleomycin, a review. *J. Med.*, **7**, 333.

EINHORN, L. & DONAHUE, J.P. (1977). Cisplatinum, vinblastine and bleomycin combination chemotherapy in disseminated testicular cancer. *Ann. Intern. Med.*, **87**, 293.

ERVIN, T.J., WEICHSELBAUM, R., MILLER, D., MESHAD, M., POSNER, M. & FABIAN, R. (1981). Treatment of advanced squamous cell carcinoma of the head and neck with cisplatin, bleomycin and methotrexate (PBM). *Cancer Treat. Rep.*, **65**, 787.

FREI, E., III, CUCCHI, C.A., ROSOWSKY, A. and 5 others (1985). Alkylating agent resistance: *in vitro* studies with human cell lines. *Proc. Natl Acad. Sci. USA*, **82**, 2158.

GLICK, J.H., MARCIAL, V., RICHTER, M. & VELEZ GARCIA, E. (1980). The adjuvant treatment of inoperable stage III and IV epidermoid carcinoma of the head and neck with platinum and bleomycin infusions prior to definitive radiotherapy: an RTOG pilot study. *Cancer*, **46**, 1919.

HAHN, G.M. & LITTLE, J.B. (1972). Plateau phase culture of mammalian cells: an *in vitro* model for human cancer. *Curr. Topics Radiat. Res. Q.*, **8**, 39.

HERMAN, T.S. & TEICHER, B.A. (1988). Platinum complexes of positively charged dyes as hyperthermia and radiosensitizing agents. *Am. Assoc. Cancer Res. Proc.*, **29**, 499.

HERMAN, T.S., TEICHER, B.A., CHAN, V., COLLINS, L.S., KAUFMANN, M.E. & LOH, C. (1988). The effect of hyperthermia on the action of *cis*-diamminedichloroplatinum(II), Rhodamine-123$_2$[tetrachloroplatinum(II)], Rhodamine-123 and potassium tetrachloroplatinate *in vitro* and *in vivo*. *Cancer Res.*, **48**, 2335.

HOLDEN, S.A., TEICHER, B.A., BOEHEIM, K., WEICHSELBAUM, R.R. & ERVIN, T.J. (1987). Platinum complexes inhibit repair of potentially lethal damage following bleomycin treatment. *Br. J. Cancer*, **55**, 245.

HONG, W.K., BHUTANI, R., SHAPSHEY, S.M. & STRONG, S. (1980). Induction chemotherapy of advanced previously untreated squamous cell head and neck cancer with cisplatin and bleomycin. In *Cisplatin: Current Status and Developments*, Prestayko, A.W., Crooke, S.T. & Carter, S.K. (eds) p. 431. Academic Press: New York.

HUBBARD, S.P., CHABNER, B.A., CANELLOS, G.P., YOUNG, R.C. & DEVITA, V.T., JR. (1975). High-dose intravenous bleomycin in treatment of advanced lymphomas. *Eur. J. Cancer*, **11**, 623.

KORBELIK, M., PALCIC, B. & SKARSGARD, L.D. (1985). Bleomycin and misonidazole cytotoxicity. *Br. J. Cancer*, **51**, 499.

LITCHFIELD, J.T. & WILCOXON, F. (1949). A simplified method of evaluating dose-effect experiments. *J. Pharmacol. Exp. Ther.*, **96**, 99.

O'HARA, J.A., DOUPLE, E.B. & RICHMOND, R.C. (1986). Enhancement of radiation-induced cell kill by platinum complexes (carboplatin and iproplatin) in V79 cells. *Int. J. Radiat. Oncol. Biol. Phys.*, **12**, 1419.

RAY, G.R., HAHN, G.M., BAGSHAW, M.A. & KURKJIAN, S. (1973). Cell survival and repair of plateau phase cultures after chemotherapy: relevance to tumor therapy and to the *in vitro* screening of new agents. *Cancer Chemother. Rep.*, **57**, 473.

RHEINWALD, J.G. & BECKETT, M.A. (1981). Tumorigenic keratinocyte lines requiring anchorage and fibroblast support cultured from human squamous cell carcinomas. *Cancer Res.*, **41**, 1657.

TALLARIDA, R.J. & MURRAY, R.B. (1981). *Manual of Pharmacologic Calculations with Computer Programs*. Springer-Verlag: New York.

TEICHER, B.A., CUCCHI, C.A., LEE, J.B., FLATOW, J.L., ROSOWSKY, A. & FREI, E. III (1986). Alkylating agents: *in vitro* studies of cross resistance patterns. *Cancer Res.*, **46**, 4379.

TEICHER, B.A. & HERMAN, T.S. (1988). Studies of CDDP and new platinum complexes for use with hyperthermia and radiation. *Radiat. Res. Soc. Proc.*, **36**, 17.

TEICHER, B.A. & HOLDEN, S.A. (1987). Antitumor and radiosensitizing activity of several platinum-positively charged dye complexes. *Radiat. Res.*, **109**, 58.

TEICHER, B.A., HOLDEN, S.A., JACOBS, J.L., ABRAMS, M.J. & JONES, A.G. (1986). Intracellular distribution of a platinum-rhodamine 123 complex in *cis*-platinum sensitive and resistant human squamous carcinoma cell lines. *Biochem. Pharmacol.*, **35**, 3365.

TEICHER, B.A., HOLDEN, S.A., KELLEY, M.J. and 5 others (1987). Characterization of a human squamous carcinoma cell line resistant to *cis*-diamminedichloroplatinum(II). *Cancer Res.*, **47**, 388.

TEICHER, B.A., ROCKWELL, S. & LEE, J.B. (1985). Radiosensitization of EMT6 cells by four platinum complexes. *Int. J. Radiat. Oncol. Biol. Phys.*, **11**, 937.

TURRISI, A.T., III, ROZENCWIEG, M., VON HOFF, D.D. & MUGGIA, F.M. (1978). The role of bleomycin in the treatment of advanced head and neck cancer. In *Bleomycin: Current Status and New Developments*, Carter, S.K., Crooke, S.T. & Umezawa, H. (eds) p. 151. Academic Press: New York.

TWENTYMAN, P.R. (1984). Bleomycin: mode of action with particular reference to the cell cycle. *Pharmacol. Ther.*, **23**, 417.

WEICHSELBAUM, R.R. (1982). The role of DNA repair processes in the response of human tumors to fractionated radiotherapy. *Int. J. Radiat. Oncol. Biol. Phys.*, **10**, 1127.

WEICHSELBAUM, R.R., DAHLBERG, W., LITTLE, J.B. and 4 others (1984). Cellular x-ray repair parameters of early passage squamous cell carcinoma lines derived from patients with known responses to radiotherapy. *Br. J. Cancer*, **49**, 595.

WEICHSELBAUM, R.R., SCHMIT, A. & LITTLE, J.B. (1982). Cellular repair factors influencing radiocurability of human malignant tumours. *Br. J. Cancer*, **45**, 10.

WITTES, R., HELLER, K., RANDOLPH, V. and 8 others (1979). *Cis*-diamminedichloroplatinum-(II)-based chemotherapy as initial treatment of advanced head and neck cancer. *Cancer Treat. Rep.*, **63**, 1533.